# Rapid reduction of malaria transmission following the introduction of indoor residual spraying in previously unsprayed districts: an observational analysis of Mopti Region, Mali, in 2017

**DOI:** 10.1186/s12936-020-03414-2

**Published:** 2020-09-19

**Authors:** Joseph Wagman, Idrissa Cissé, Diakalkia Kone, Seydou Fomba, Erin Eckert, Jules Mihigo, Elie Bankineza, Mamadou Bah, Diadier Diallo, Christelle Gogue, Kenzie Tynuv, Andrew Saibu, Jason H. Richardson, Christen Fornadel, Laurence Slutsker, Molly Robertson

**Affiliations:** 1 PATH, Washington, DC USA; 2 Programme National de Lutte contre le Paludisme, Bamako, Mali; 3 RTI International, Washington, DC USA; 4 PMI, USAID, Bamako, Mali; 5 Abt Associates, Bamako, Mali; 6 MEASURE Evaluation, Bamako, Mali; 7 IVCC, Accra, Ghana; 8 IVCC, Washington, DC USA

**Keywords:** Indoor residual spraying, Malaria incidence, Next-generation IRS, Observational impact analysis

## Abstract

**Background:**

The National Malaria Control Programme (NMCP) of Mali has had recent success decreasing malaria transmission using 3rd generation indoor residual spraying (IRS) products in areas with pyrethroid resistance, primarily in Ségou and Koulikoro Regions. In 2015, national survey data showed that Mopti Region had the highest under 5-year-old (u5) malaria prevalence at 54%—nearly twice the national average—despite having high access to long-lasting insecticidal nets (LLINs) and seasonal malaria chemoprevention (SMC). Accordingly, in 2016 the NMCP and other stakeholders shifted IRS activities from Ségou to Mopti. Here, the results of a series of observational analyses utilizing routine malaria indicators to evaluate the impact of this switch are presented.

**Methods:**

A set of retrospective, eco-observational time-series analyses were performed using monthly incidence rates of rapid diagnostic test (RDT)-confirmed malaria cases reported in the District Health Information System 2 (DHIS2) from January 2016 until February 2018. Comparisons of case incidence rates were made between health facility catchments from the same region that differed in IRS status (IRS vs. no-IRS) to describe the general impact of the 2016 and 2017 IRS campaigns, and a difference-in-differences approach comparing changes in incidence from year-to-year was used to describe the effect of suspending IRS operations in Ségou and introducing IRS operations in Mopti in 2017.

**Results:**

Compared to communities with no IRS, cumulative case incidence rates in IRS communities were reduced 16% in Ségou Region during the 6 months following the 2016 campaign and 31% in Mopti Region during the 6 months following the 2017 campaign, likely averting a total of more than 22,000 cases of malaria that otherwise would have been expected during peak transmission months. Across all comparator health facilities (HFs) where there was no IRS in either year, peak malaria case incidence rates fell by an average of 22% (CI_95_ 18–30%) from 2016 to 2017. At HFs in communities of Mopti where IRS was introduced in 2017, peak incidence fell by an average of 42% (CI_95_ 31–63%) between these years, a significantly greater decrease (p = 0.040) almost double what was seen in the comparator HFCAs. The opposite effect was observed in Ségou Region, where peak incidence at those HFs where IRS was withdrawn after the 2016 campaign increased by an average of 106% (CI_95_ 63–150%) from year to year, also a significant difference-in-differences compared to the comparator no-IRS HFs (p < 0.0001).

**Conclusion:**

Annual IRS campaigns continue to make dramatic contributions to the seasonal reduction of malaria transmission in communities across central Mali, where IRS campaigns were timed in advance of peak seasonal transmission and utilized a micro-encapsulated product with an active ingredient that was of a different class than the one found on the LLINs used throughout the region and to which local malaria vectors were shown to be susceptible. Strategies to help mitigate the resurgence of malaria cases that can be expected should be prioritized whenever the suspension of IRS activities in a particular region is considered.

## Background

Although Mali remains a highly malaria-endemic country, with an estimated 7.2 million cases in 2017 and 16.9 million people living in high-risk communities [1], the National Malaria Control Programme (NMCP) has recently demonstrated success in reducing morbidity and mortality: between 2013 and 2017 malaria deaths fell by an estimated 38% [1, 2] and nationwide malaria prevalence in children under 5 years old (u5) dropped from 52 to 32% [3, 4]. Helping to drive these reductions is the multifaceted National Malaria Control Strategic Plan, implemented by the NMCP with support from several partner organizations [5]. One of the key elements of the strategic plan is malaria vector control [6], which includes the goal of universal coverage of the population at risk (18.5 million; 100% of Mali’s population) with access to a long-lasting insecticidal net (LLIN), complimented by indoor residual spraying (IRS) in certain high-risk districts across the country. The LLIN strategy employs rolling mass LLIN distribution campaigns, organized every 3 years at a regional level, as well as routine distribution of LLINs to pregnant women and children visiting public health clinics for antenatal care and routine childhood immunization visits. The IRS strategy has been mostly implemented through a close collaboration with the US President’s Malaria Initiative (PMI) Africa Indoor Residual Spraying (AIRS) project (now the VectorLink programme).

Until 2010, the PMI AIRS project in Mali focused on spraying pyrethroid insecticides in several high-burden districts of Koulikoro and Ségou Regions. In 2011, concerns about the emergence and spread of pyrethroid resistance in the main malaria vectors of Mali (the *Anopheles gambiae* species complex) prompted a switch in IRS active ingredients from pyrethroids to the carbamate insecticide bendiocarb [7]. Beginning in 2014, the programme transitioned from using bendiocarb to a third-generation indoor residual spray product (3GIRS; insecticide formulations that are effective at controlling pyrethroid-resistant mosquitoes and have a target residual efficacy of 6 months)—a microencapsulated formulation of the organophosphate insecticide pirimiphos-methyl (PM) (Actellic^®^ 300CS; Syngenta A.G., Basel, Switzerland) [8]. Recent studies using routine surveillance data from Ségou Region have shown that the IRS campaigns from 2012 to 2015, which utilized bendiocarb and PM to spray more than 200,000 structures each year, were good public health investments—protecting more than 500,000 people a year for around US$7.00 a person while simultaneously reducing indoor resting densities of *An. gambiae* sensu lato (*s.l*.), by around 80%, and passively reported confirmed malaria cases, by around 33% [9, 10].

By 2015, though, national survey data showed that Mopti Region had the highest u5 malaria prevalence at 53.4%—nearly twice the national average and significantly greater than Ségou (21.9%)—despite having high access to LLINs and seasonal malaria chemoprevention (SMC) [4]. Additionally, migration of displaced people to Mopti from northern Mali, where malaria transmission is substantially lower and acquired immunity is thought to be low, was further complicating the malaria control situation in the region [11]. Accordingly, in 2016 a decision to shift IRS activities from Barouéli District in Ségou Region to the districts of Mopti, Bandiagara, Bankass, and Djenné in Mopti Region was made by the NMCP and other stakeholders [12].

Here, a retrospective, observational time series analysis of monthly malaria incidence rates reported by health facilities in Ségou and Mopti Regions from January 2016 to February 2018 is used to estimate the epidemiological impact of (1) introducing IRS into parts of four previously unsprayed districts in Mopti and (2) suspending IRS operations in the Barouéli District of Ségou.

## Methods

### Study design

A set of retrospective, observational (ecological), time-series analyses were performed using monthly incidence rates of rapid diagnostic test (RDT)-confirmed malaria cases reported in the District Health Information System 2 (DHIS2) from January 2016 until February 2018. Monthly case numbers were aggregated at the health facility level for clinics in the districts of Ségou and Mopti Regions. Baseline population estimates, based on 2012 census results, for each health facility catchment area (HFCA) were obtained from the *Ministère de la Santé de la République du Mali* (Ministry of Health), *Direction Régionale de la Santé*. IRS programme implementation data from the PMI AIRS project [13, 14] was used to stratify analyses by HFCA spray status, either IRS or non-IRS.

### Study districts

**Fig. 1:**
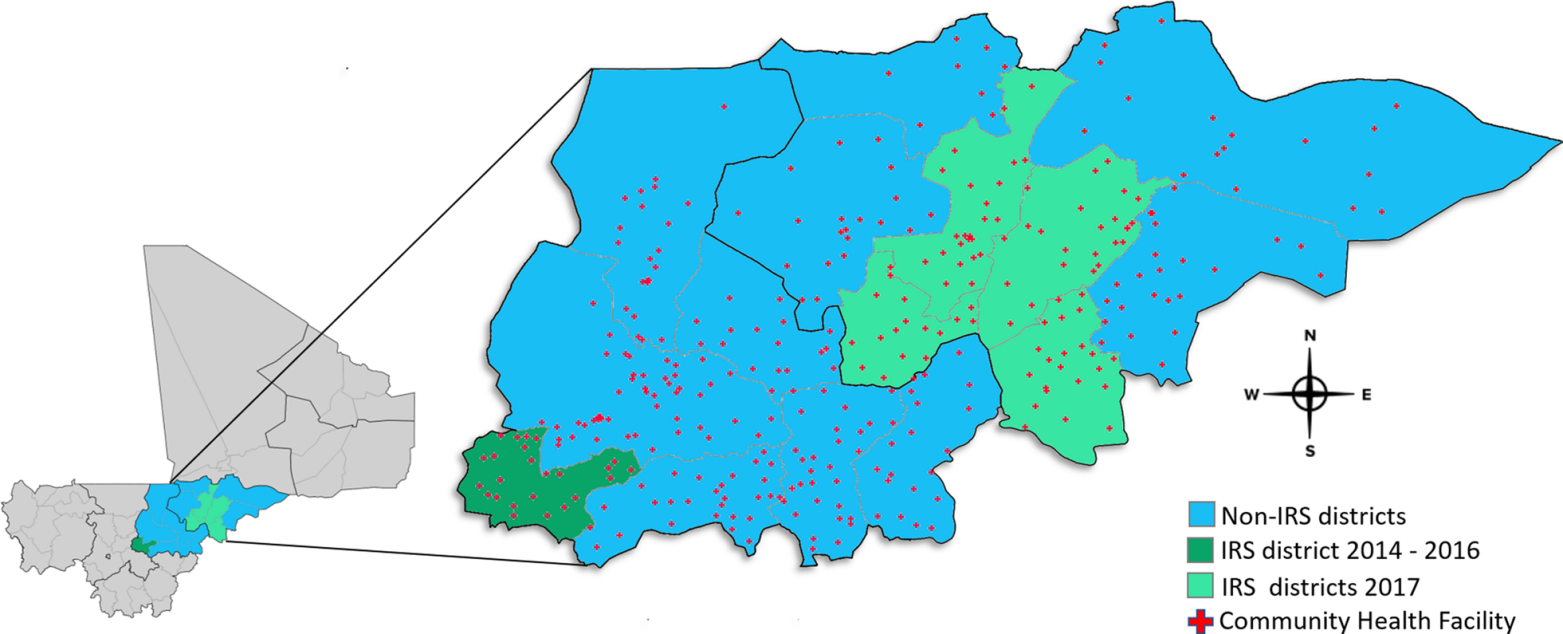
The location of the study districts, with intervention status indicated. After the 2016 indoor residual spraying (IRS) campaign, spray operations were shifted from Barouéli District in Ségou Region (dark green) to the districts of Mopti, Bandiagara, Bankass, and Djenné in Mopti Region (light green)

The districts included in this report are shown in Fig. 1 and Table 1. Ségou and Mopti contain 15 administrative districts (*cercles*) covering roughly 145,000 km^2^, with an estimated 2017 population of around 5.8 million—representing about 30% of the total population of Mali [15]. The malaria burden is relatively high in the two regions (2015 Malaria Indicator Survey results estimated an u5 prevalence of 22% in Ségou and 54% in Mopti) [4], and transmission is seasonal—typically highest from August through November and coinciding with the rainy season, when *An. gambiae* sensu stricto (*s.s*.), *Anopheles coluzzii*, and *Anopheles arabiensis* are all present [9]. The most recent LLIN mass distribution campaigns had been completed in 2015 in Ségou [12] and 2017 in Mopti [6], and both regions have benefitted from continuous routine LLIN distribution through antenatal care and childhood immunization visits since at least 2013 [6]. During the study period, consistently high LLIN ownership (90–95% of households owned at least one LLIN) and use (83–86% of children u5 used an LLIN last night) were reported [16]. Local vector populations were also reported to be highly resistant to pyrethroids and dichlorodiphenyltrichloroethane but susceptible to carbamates and organophosphates throughout the study period: mortality of local vector populations exposed to pyrethroids in standard diagnostic WHO tube tests ranged from 18 to 55% against permethrin and from 45 to 77% against deltamethrin, while CDC bottle bioassays indicated that resistance remained against both chemicals at up to 10× the diagnostic dose [9, 17]. Vector populations remained 100% susceptible to PM throughout the study period [9]. Both regions have also participated in annual SMC campaigns since 2015 [18] and benefited from access to intermittent preventive treatment in pregnant women, free of cost to all residents. Across all study districts, the SMC campaigns targeted all resident children aged 3 to 59 months with a monthly course of sulfadoxine-pyrimethamine plus amodiaquine for 4 months of the rainy season, beginning each August. District-level coverages obtained by each SMC campaign were comparable during the study period and are presented in Table 1. Additionally, the districts of Ségou and Mopti have been shown to be similar to one another with respect to population density, rainfall patterns, malaria transmission seasonality, and population-adjusted *Plasmodium* prevalence rates [19], further strengthening the case to use the non-IRS districts and HFCAs as time- and climate-matched comparator districts for these observational studies.

**Table 1:** Summary of the malaria control landscape in the study districts

Region	District	2016							2017						
		IRS							IRS						
		AI	Number of Structures Sprayed	Acceptance Rate^a^	No. of HFCA Targeted (%)^b^	Population Protected^c^	LLINs Distributed	SMC^d^	AI	Number of Structures Sprayed	Acceptance Rate^a^	No. of HFCA Targeted (%)^b^	Population Protected^c^	LLINs Distributed	SMC^d^
Ségou	Barouéli	Actellic	71,333	97%	19/19 (100%)	279,135	ANC/EPI	68%	None	–	–	–	–	ANC/EPI	> 80%
	Bla	None	–	–	–	–	ANC/EPI	99%	None	–	–	–	–	ANC/EPI	> 80%
	Macina	None	–	–	–	–	ANC/EPI	100%	None	–	–	–	–	ANC/EPI	> 80%
	Segou	None	–	–	–	–	ANC/EPI	87%	None	–	–	–	–	ANC/EPI	> 80%
	Niono	None	–	–	–	–	ANC/EPI	93%	None	–	–	–	–	ANC/EPI	> 80%
	San	None	–	–	–	–	ANC/EPI	81%	None	–	–	–	–	ANC/EPI	> 80%
	Tominian	None	–	–	–	–	ANC/EPI	97%	None	–	–	–	–	ANC/EPI	> 80%
Mopti	Bandiagara	None	–	–	–	–	ANC/EPI	99%	Actellic	91,291	95%	17/24 (70%)	302,671	Universal	> 80%
	Bankass	None	–	–	–	–	ANC/EPI	100%	Actellic	25,917	96%	6/24 (23%)	84,929	Universal	> 80%
	Djenné	None	–	–	–	–	ANC/EPI	83%	Actellic	35,173	97%	7/17 (41%)	138,447	Universal	> 80%
	Douentza	None	–	–	–	–	ANC/EPI	60%	None	–	–	–	–	Universal	> 80%
	Koro	None	–	–	–	–	ANC/EPI	100%	None	–	–	–	–	Universal	> 80%
	Mopti	None	–	–	–	–	ANC/EPI	80%	Actellic	75,265	93%	19/26 (74%)	297,154	Universal	> 80%
	Ténenkou	None	–	–	–	–	ANC/EPI	100%	None	–	–	–	–	Universal	> 80%
	Youwarou	None	–	–	–	–	ANC/EPI	100%	None	–	–	–	–	Universal	> 80%

*AI* Active ingredient, *ANC* antenatal clinic, *EPI* expanded program on immunization, *HFCA* health facility catchment area, *IRS* indoor residual spray, *LLIN* long lasting insecticidal nets, *SMC* seasonal malaria chemoprevention, *SP *+ *AQ* sulfadoxine-pyrimethamine + amodiaquine

^a^ % of structures targeted for IRS that were sprayed

^b^Number of health facility catchment areas that received IRS. In Barouéli in 2016 a blanket spraying approach targeted all houses in each HFCA

in Mopti in 2017 a more focal spraying approach targeted all houses in select HFCAs

^c^Total number of residents of houses that were sprayed

^d^ % of target population receiving at least 4 courses of SMC with SP + AQ; 2017 data awaiting final validation

### IRS intervention

IRS was implemented by the NMCP with support of the PMI AIRS project in Barouéli District of Ségou Region in 2016, and in the districts of Mopti, Bandiagara, Bankass, and Djenné in Mopti Region in 2017 [13, 14]. PM (Actellic^®^300CS) was used in both campaigns. The 2016 campaign lasted from July 9 to August 12 and sprayed a total of 71,333 structures out of 73,528 eligible structures encountered (a 97% acceptance rate; Table 1) in Barouéli District, using a blanket spraying approach that aimed to spray every eligible house in the district. The 2017 campaign in the four districts of Mopti Region lasted from July 24 until August 27, and utilized a slightly different approach; only a certain percentage of HFCAs that were easily accessible, given safety and logistical considerations, were targeted for spraying in each district. The percentage of HFCAs that were targeted varied by district: 74% in Mopti, 70% in Bandiagara, 23% in Bankass, and 41% in Djenné. Within each selected catchment area, all eligible houses were targeted. Overall, the 2017 campaign sprayed 239,350 houses across the four districts, with an average acceptance rate of 96% (Table 1). In both campaigns, eligible houses were structures with one or more rooms used for sleeping and interior wall surfaces that were sprayable. Entomological monitoring from 2017 indicated that Actellic had a residual efficacy of 3–4 months, depending on wall type, as measured with standard WHO cone bioassays [9].

### Estimation of malaria case incidence rates

From January 2016 to February 2018, the DHIS2 recorded a total of 11,280 monthly malaria reports from 314 public community health facilities (*Centre de santé communautaires,* CSCom) across Ségou and Mopti. Only surveillance of cases from public health facilities, where national malaria case management guidelines are to test all suspected cases with an RDT, are included in the present analysis. A total of 497,498 RDT-confirmed cases of *Plasmodium falciparum* malaria were reported in u5 children, out of 700,767 suspected cases (patients with a fever) tested, for an overall u5 test positivity rate of 71%. Almost two-thirds (64%) of all confirmed cases were reported during the months August to January, corresponding to seasonal rainfall. During this period, 93% of all suspected cases reporting to the health system in Ségou and Mopti received a diagnosis by RDT and/or microscopy, and 94% of confirmed cases were treated with artemisinin-based combination therapy. No substantial variation in these measures were observed across districts (82–99% tested; 86–100% treated) or months (86–97% tested; 87–100% treated). District reporting rates were greater than 98% for both IRS and non-IRS districts, though district-months in which no data were reported were excluded from analysis. Additionally, since 2015, MEASURE Evaluation has actively assisted the Ministry of Health with DHIS2 data quality assurance activities at all levels of the system, including tracking commodity stockouts, which were minor during the period analysed here. Monthly malaria incidence rates were calculated by dividing the appropriate number of RDT + test results recorded in the DHIS2 database by the corresponding HFCA population denominators [15].

### General analysis of the impact of IRS on incidence rates: 2016 to 2017

For this analysis of monthly trends in malaria incidence in the u5 population, a quasi-experimental time series approach was used. The regions of Ségou and Mopti were analysed separately; for each analysis, health facilities were stratified by IRS status (IRS and non-IRS) based on data compiled from PMI AIRS End of Spray Reports and national Malaria Operational Plans (http://www.pmi.gov/where-we-work/mali). To estimate the impact of IRS, the cumulative incidence of RDT-positive malaria cases observed during the 6 months following each IRS campaign (which also corresponds to the high transmission season) was calculated for health facilities that received IRS and compared to the cumulative malaria incidence observed in health facilities from the same region that did not receive IRS. The total number of cases averted during each post-IRS window was calculated by multiplying the observed cumulative incidence reduction by the corresponding strata population estimates.

### Estimating the impact of introducing IRS operations into Mopti Region, 2017

A difference-in-differences approach was used to compare changes in malaria incidence observed at individual health facilities, grouped by IRS status, from year to year to estimate the impact of introducing IRS operations in Mopti Region in 2017. In short, monthly u5 malaria incidence rates were calculated for each health facility in Mopti that benefited from IRS (51 facilities) and each that did not (117 facilities). Changes in the cumulative u5 incidence from the 2016 transmission season to the 2017 transmission season were calculated and mapped. Lastly, the average change in incidence observed at regional health facilities that received IRS beginning in 2017 was compared to the average change in incidence observed at the health facilities that did not receive IRS in either year, using Student’s *t* test at α = 0.05.

### Estimating the impact of suspending IRS operations in Ségou Region, 2017

The same difference-in-differences approach was used to quantify the impact of suspending IRS operations after the 2016 spray campaign in the Barouéli District of Ségou Region. The average change in incidence from the 2016 to 2017 transmission seasons observed at the 19 health facilities in Barouéli was compared to the average change observed at the health facilities in the rest of the districts of Ségou Region (121 health facilities), which had not received IRS in either year.

### Data analysis and visualization

Datasets were organized, cleaned, transformed, and joined using Microsoft Excel 2013 with Power Query v2.41 (Microsoft Corporation; Redmond, WA) and Tableau v10.0 (Tableau Software Incorporated; Seattle, WA). Descriptive statistics were calculated using Excel 2013 and Tableau v10.0. Confidence intervals were calculated and Student’s t-tests comparing averaged incidence rates of malaria across districts by IRS status were conducted using STATA SE 14.2 (StataCorp; College Station, TX). Geographical Information System analysis and mapping were performed using QGIS v2.16 [20]. Shapefiles were downloaded from the GADM database of Global Administrative Areas [21] in December 2017.

## Results

### General impact of IRS campaigns in 2016 and 2017

**Fig. 2:**
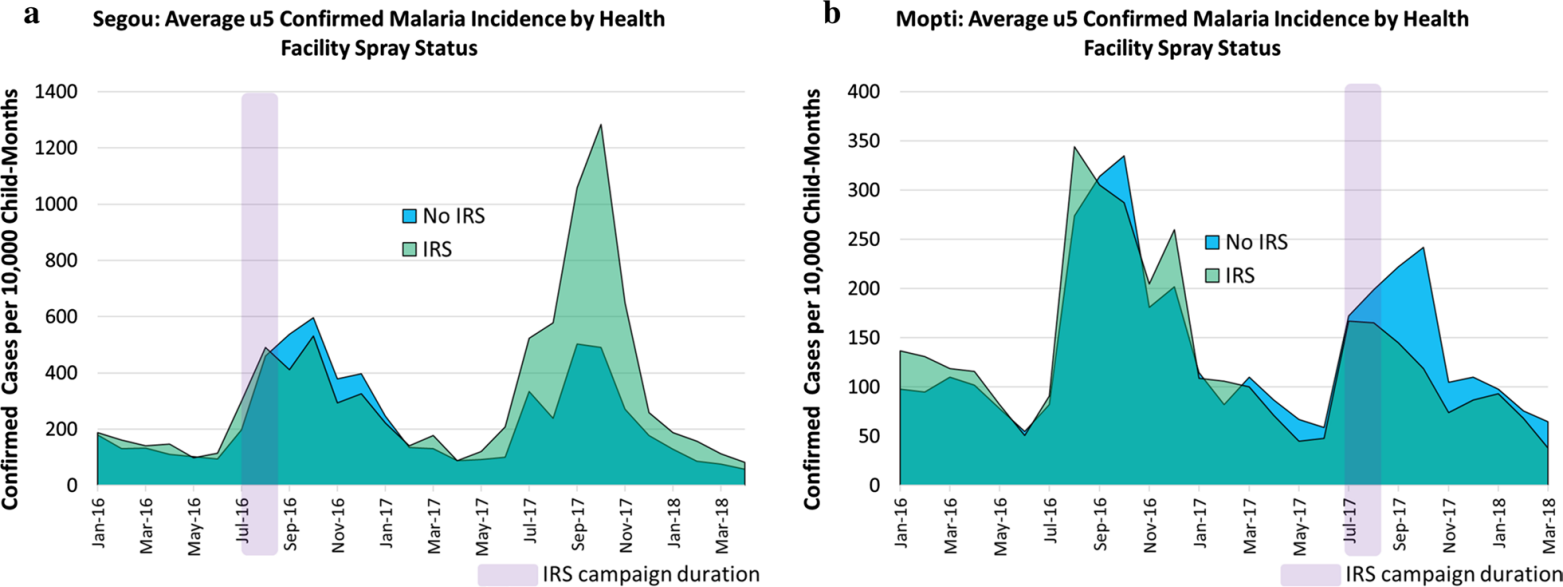
General trends in monthly confirmed case incidence rates. **a** Ségou and **b** Mopti regions of Mali, 2016–2017. Illustrated are direct comparisons of the incidence rates observed at health facilities whose populations benefited from indoor residual spraying (IRS) in either year (green incidence curves) versus incidence rates observed at comparator non-IRS health facilities from the same region

The average monthly incidence of RDT-confirmed cases in the u5 population, by HFCA IRS status, are presented for Ségou Region (2016 IRS campaign) and Mopti Region (2017 IRS campaign) in Fig. 2. Considering the cumulative case incidence from the six high-transmission months following the IRS campaign (September to February), the effect of IRS in Ségou in 2016 was comparatively modest but consistent: the observed case incidence at IRS health facilities was reduced by 16% in comparison to non-IRS facilities (Fig. 2a), which translates into around 5200 u5 cases averted in Barouéli that year. The observed impact of the 2017 campaign in Mopti was almost double that observed in Ségou the year prior, with case incidence rates reduced by 31% at IRS health facilities in comparison to non-IRS health facilities (Fig. 2b)—an estimated 17,500 fewer cases than expected in the u5 population.

### The impact of changing IRS status after the 2016 campaign

**Fig. 3:**
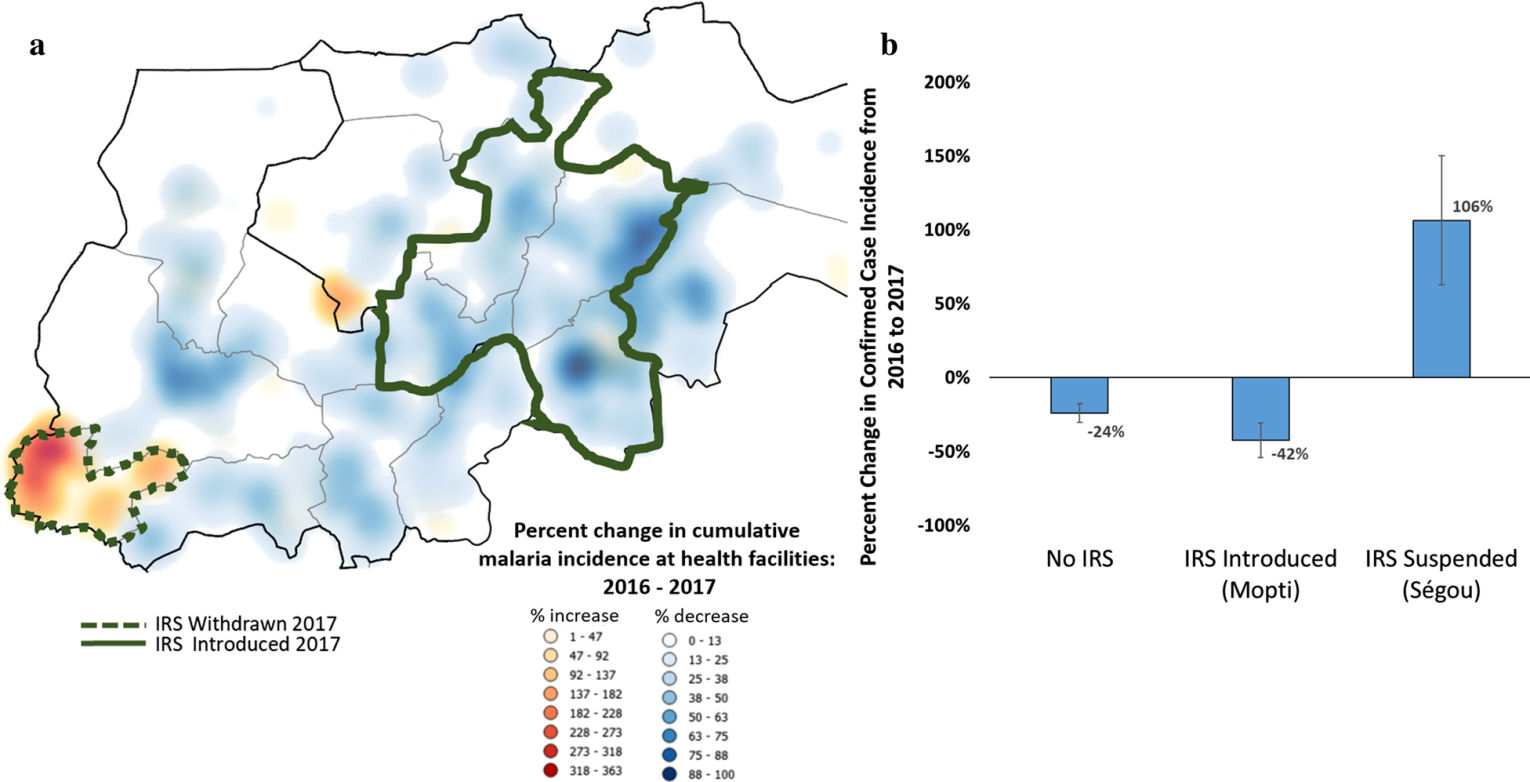
Results of the difference-in-differences analyses. **a** How peak (September–February) malaria case incidence rates changed from 2016 to 2017 at the health facility level in districts where indoor residual spraying (IRS) was removed (dotted line border), districts where IRS was introduced (solid green border), and the remaining comparator districts where there was no IRS in either year. At each health facility, blue indicates a drop in malaria incidence and red indicates an increase, and the intensity of the colour represents the magnitude of the change. **b** The results of Student’s t-tests comparing the rates of changing incidence across the two IRS scenarios

While the reductions in incidence observed at health facilities within the IRS areas of Ségou in 2016 and Mopti in 2017 correspond both temporally and geographically with the PMI AIRS spray operations, the strength of the association can be further evaluated with a difference-in-differences approach—taking advantage of the fact that the intervention status of the IRS health facilities changed from year to year in both regions, as shown in Fig. 3.

Across all comparator health facilities where there was no IRS in either year (n = 238), peak malaria case incidence rates (September to February) fell by an average of 22% (CI_95_ 18–30%) from 2016 to 2017 (Fig. 3). In those HFCAs of Mopti where IRS was introduced in 2017 (n = 51), peak incidence fell by an average of 42% (CI_95_ 31–63%), a significantly greater decrease (p = 0.040) more than double what was seen in the comparator HFs (Fig. 3). The opposite effect was observed in Ségou Region, where peak incidence at those HFs where IRS was withdrawn after the 2016 campaign (n = 19) increased by an average of 106% (CI_95_ 63–150%) from year to year, also a highly significant difference-in-differences compared to the standard no-IRS HFs (p < 0.0001) (Fig. 3).

## Discussion

Vector control, primarily the widespread use of LLINs and targeted use of IRS, is widely recognized as one of the primary drivers of the substantial reduction in global malaria burden that has been observed since 2000 [1, 22] and is widely recognized as a critical component of global efforts to further control, and eventually eliminate, malaria [23]. Nonetheless, the emergence, intensification, and rapid spread of insecticide resistance in malaria vectors (in combination with many other ecological and systemic factors) is increasing the complexity of the global malaria vector control landscape and driving the need for new tools and innovative approaches [23, 24]. One set of questions that frequently arises in this environment asks how, when, and where to most effectively add IRS to a malaria control programme that already uses strategies to support universal coverage of populations at risk with LLINs.

The present study investigated the impact of IRS with Actellic^®^300CS, a 3GIRS product, in communities of central Mali where levels of pyrethroid-only LLIN ownership (90–95% of households own at least one LLIN) and use (more than 80% of children u5 are reported to have slept under an LLIN) were reported to be high [16], SMC coverage in the u5 population was consistent in study districts across both study years (Table 1), and the primary vector mosquitoes were resistant to pyrethroid insecticides [9, 17]. Results show that the IRS campaigns of 2016 and 2017 were associated with reduced malaria transmission in the u5 population, clearly indicating a positive incremental impact of IRS with a non-pyrethroid insecticide used in combination with standard, pyrethroid-only LLINs. The general analysis of monthly incidence trends comparing rates of confirmed cases passively reported in the DHIS2 from IRS and non-IRS communities is compelling, suggesting protective effects ranging from 16 to 31% depending on the region sprayed and the year (Fig. 2).

The positive impact of IRS is in these communities is emphasized when looking at differences in how peak transmission rates changed from year to year as the location of IRS operations shifted from 2016 to 2017. In HFCAs of Mopti where IRS was newly introduced in 2017, malaria incidence fell almost twice as much as in comparable HFCAs that did not receive IRS in either year. Conversely, in HFCAs of Ségou where IRS was suspended prior to the 2017 spray season, malaria incidence increased substantially compared to similar HFCAs that did not receive IRS in either year, where a general trend of modestly decreasing malaria case incidence was observed (Fig. 3). Of note, these seasonal difference-in-differences results from Ségou were observed 3 years after the previous LLIN universal coverage campaign at a time when those nets were likely near the end of their target residual effectiveness. In Mopti, however, the introduction of IRS occurred in the same year as a successful universal LLIN coverage campaign. Though it is not possible to estimate the overall impact of LLINs (or how this impact may have waned over time as the nets aged) with the data analysed here, the positive impact of IRS evident in both regions suggests an incremental impact of IRS in addition to whatever baseline level of control was achieved by the pyrethroid-only LLINs also present.

The ecological studies presented here take advantage of some natural comparisons that arose as the result of normal operational decisions made by the NMCP in Mali. As such, there are many important limitations to the study, including its observational, non-randomized design. To help further ensure good comparability between intervention (IRS) and non-intervention (non-IRS) HFCAs evaluated here, IRS facilities from Ségou were compared only to non-IRS facilities also from Ségou. Similarly, intervention facilities from Mopti were compared only to non-intervention facilities also from Mopti. This step was important not only as an attempt to enhance the similarity between study arms in terms of population, socio-economic, cultural, and ecological factors, but also because direct comparisons of passive surveillance data across the two regions are challenging. Ségou and Mopti Regions have dramatically different health service utilization rates: based on outpatient visits per capita reported in the DHIS2 during this study period, a resident of Mopti was around 30% less likely to visit a health centre during the year than a resident of Ségou (2460 visits per 10,000 per year vs. 3500 visits per 10,000 per year).

Another limitation of the study is that the influx of migrants from more Northern Districts into the underlying study population of Mopti complicates the estimation of case incidence rates in those districts. Though this influx began before the study period and was not known to be skewed between IRS and non-IRS districts, an overall increase in the baseline population of Mopti from the estimates based on the 2012 census could have led to an over-estimation of crude incidence rates across the province in both years. Also, safety and accessibility were used to help prioritize which HFCAs received IRS in Mopti in 2017. It is possible that these same factors might have influenced differences in healthcare-seeking behaviours across IRS and non-IRS HFCAs. If residents of more insecure, less accessible non-IRS communities were also less likely to engage in healthcare-seeking then the case incidence rates from those HFCAs could be under-estimated, and the overall impact of IRS estimated from the routine surveillance data analysed here could also be under-estimated.

These limitations make direct comparisons of health facility case numbers between regions very difficult to interpret, and therefore comparisons of the cost-effectiveness of IRS across the regions potentially misleading. Though it is tempting to ask whether the net benefit, in terms of health system cases averted, of introducing IRS into new districts of Mopti was counterbalanced by the increase in cases observed after suspending IRS in Ségou, given these limitations this question is beyond the scope of the present analysis. What is clearly evident, however, is that IRS continues to make demonstrable positive contributions to malaria control throughout central Mali, where malaria vectors are resistant to pyrethroids, LLIN access and use are high, and access to seasonal malaria chemoprevention has rapidly expanded in recent years. Further work is ongoing to describe the impact of these IRS campaigns on key entomological indicators from the same study areas [25], which should lead to a broader understanding of the overall effect of IRS on the reduction of malaria transmission in central Mali.

## Conclusions

Annual IRS campaigns continue to make dramatic contributions to the seasonal reduction of malaria transmission in communities across central Mali, where LLIN access and use are high and SMC is also successfully co-implemented. While attempting to apply these lessons to other transmission settings, it is important to note that the successful campaigns evaluated here (1) were timed in advance of peak seasonal transmission and (2) utilized a microencapsulated product with an active ingredient that was of a different class than the one found on the LLINs used throughout the region, and to which local malaria vectors were shown to be susceptible.

Though decisions about precisely where to implement IRS as part of an integrated malaria control programme remain complex, the results presented here add to an expanding evidence base that supports the value of IRS with non-pyrethroid insecticides in communities with moderate to high transmission, abundant pyrethroid-only LLINs, and pyrethroid-resistant vectors. As such, it is easy to envision a continued role for IRS across much of Africa moving forward, though the annual sustainability of such efforts must be considered. Also, if suspension of IRS activities in a particular region is considered, strategies to help mitigate the resurgence of malaria cases that can be expected should be prioritized. In addition, exactly where, when, and how to maximize the impact and cost-effectiveness of next-generation IRS as part of integrated malaria control programmes will require continual re-evaluation, especially as the LLIN landscape rapidly changes to incorporate the use of novel, next-generation bed nets.

## Data Availability

Intervention, entomology, and climate datasets used and/or analysed during this study were consolidated from public sources and are available from the corresponding author upon reasonable request. Requests about the malaria surveillance datasets analysed here should be directed to IC (idrissaciss68@yahoo.fr): Director, Programme National de Lutte contre le Paludisme, Bamako, Mali.

## Abbreviations

3GIRs: Third generation indoor residual spray
AIRS: Africa indoor residual spraying
CI_95_: 95% confidence interval
DHIS2: District health information system 2
HF: Health facility
HFCA: Health facility catchment area
IRS: Indoor residual spray
LLIN: Long lasting insecticidal net
NMCP: National malaria control programme
PMI: US President’s Malaria Initiative
RDT: Rapid diagnostic test
SMC: Seasonal malaria chemoprevention
u5: Children under 5 years old
